# Influence of Small Quantities of Water on the Physical Properties of Alkylammonium Nitrate Ionic Liquids

**DOI:** 10.3390/ijms22147334

**Published:** 2021-07-08

**Authors:** David Ausín, Juan J. Parajó, José L. Trenzado, Luis M. Varela, Oscar Cabeza, Luisa Segade

**Affiliations:** 1 Departamento de Física, Facultade de Ciencias, Campus da Zapateira, Universidade da Coruña, 15071 A Coruña, Spain; david.ausin.neira@udc.es (D.A.); oscar.cabeza@udc.es (O.C.); 2 Grupo de Nanomateriais, Fotónica e Materia Branda, Departamento de Física de Partículas y Departamento de Física Aplicada, Universidade de Santiago de Compostela, Campus Vida s/n, 15782 Santiago de Compostela, Spain; juanjose.parajo@usc.es (J.J.P.); luismiguel.varela@usc.es (L.M.V.); 3 Departamento de Química e Bioquímica, CIQUP-Centro de Investigaçao em Química da Universidade do Porto, Universidade do Porto, P-4169-007 Porto, Portugal; 4 Departamento de Física, Universidad de Las Palmas de Gran Canaria, 35017 Las Palmas Gran Canaria, Spain; jose.trenzado@ulpgc.es

**Keywords:** ionic liquid, ethylammonium nitrate, propylammonium nitrate, water-free, density, viscosity, electrical conductivity, refractive index, surface tension

## Abstract

This paper presents a comprehensive study of two alkylammonium nitrate ionic liquids. As part of this family of materials, mainly ethylammonium nitrate (EAN) and also propylammonium nitrate (PAN) have attracted a great deal of attention during the last decades due to their potential applications in many fields. Although there have been numerous publications focused on the measurement of their physical properties, a great dispersion can be observed in the results obtained for the same magnitude. One of the critical points to be taken into account in their physical characterization is their water content. Thus, the main objective of this work was to determine the degree of influence of the presence of small quantities of water in EAN and PAN on the measurement of density, viscosity, electrical conductivity, refractive index and surface tension. For this purpose, the first three properties were determined in samples of EAN and PAN with water contents below 30,000 ppm in a wide range of temperatures, between 5 and 95 °C, while the last two were obtained at 25 °C. As a result of this study, it has been concluded that the presence of water is critical in those physical properties that involve mass or charge transport processes, resulting in the finding that the absolute value of the average percentage change in both viscosity and electrical conductivity is above 40%. Meanwhile, refractive index (≤0.3%), density (≤0.5%) and surface tension (≤2%) present much less significant changes.

## 1. Introduction

During the last decades, the study of ionic liquids (ILs) has aroused enormous interest in the scientific community because of their extraordinary physical properties and versatility, which have made them worthy of becoming a true field of research [1]. The main feature that made them attractive is that they can be tailor-made by combining the cations and anions of which they can be composed, obtaining a great variety of ILs with very different properties and applications.

After a couple of decades of extensive development in the study of the potential uses of ILs, their applications are now a reality in many branches of science and industry. Thus, their uses have been demonstrated in a wide range of areas of analytical chemistry [2], organic chemistry [3,4], electrochemical conversion and energy storage [5], biosensing technology [6], pharmaceutical and biomedical industry [7,8] or recovery of industrial solvents [9,10], to name but a few. In this context, the characterization of these materials through experimental, theoretical or computational methods plays a decisive role to promote their industrial applications [11].

**Figure 1:**
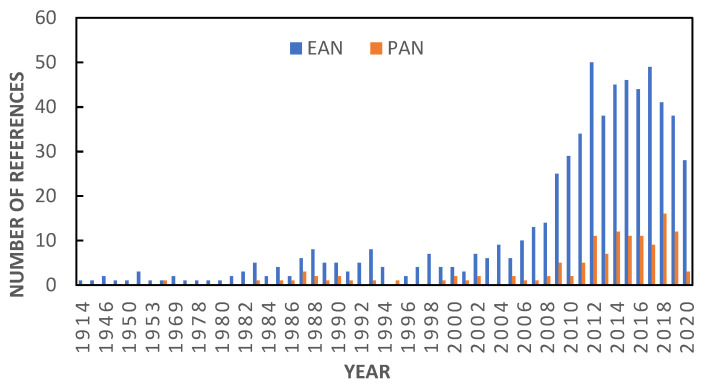
Increase in the number of bibliographic references related to EAN and PAN from their discovery to nowadays (SciFinder^n^ database).

In this work, we continue with the experimental study of ILs to which our groups have been devoted in the past years [12,13,14], and specifically, we focus on one of our lines of work dedicated to protic ionic liquids (PILs) [15,16], the usefulness of which has been widely tested. Within this class of materials, two representatives of the family of alkylammonium nitrates, ethylammonium nitrate (EAN) and propylammonium nitrate (PAN), have attracted great attention as a result of their multiple applications. Many examples of them can be found in fields as diverse as electrodeposition, electrochemical exfoliation, liquid–liquid extraction, organic or inorganic synthesis, biocatalysis, lubrication or biological medium solvents, among others [17,18]. More recent works have already explored their utility as components of smart materials [19], in hydrogen sorption processes [20], in thermoelectric generator devices [21] or in studies on the structural stability and aggregation state of proteins [22]. In general, EAN and PAN have been the subject of many studies, reported in more than 700 bibliographic references (SciFindern database), as can be seen in Figure 1. A large part of these references has been devoted to exploring the uses of these compounds, while approximately half of them have focused on the determination of their physicochemical properties.

Considering that a thorough and accurate characterization of these materials has a direct impact on the knowledge of their structure and in the design of their potential applications, we have performed an exhaustive compilation of some of the most relevant physical properties. Thus, we have reviewed the state of the art of density, viscosity, electrical conductivity, refractive index and surface tension for EAN and PAN at different temperatures [23,24,25,26,27,28,29,30,31,32,33,34,35,36,37,38,39,40,41,42,43,44,45,46,47,48,49,50,51,52,53,54,55,56,57,58,59,60,61,62,63,64,65,66,67,68,69,70,71,72,73,74,75,76,77,78,79,80,81,82,83,84,85,86,87,88,89].

These properties enable us to anticipate the suitability of an IL in some applications. For instance, density and refractive index are two remarkably interesting parameters because, besides being useful to identify a substance, they allow obtaining other information such as ionic conductivities or electronic polarizabilities, respectively. Viscosity could be related to extraction processes, either facilitating dispersion (low viscosity) or by avoiding losses (high viscosity) [2]. In the case of batteries or electrochemical sensing systems, it is essential information to know thermal stability, electrochemical stability, electrical conductivity and viscosity [5]. Other studies that have analyzed the ability of certain ILs to act as surfactants have made use of the information provided by surface tension data, with the aim of developing drug delivery systems [8]. In all cases, it must be taken into account that the properties tend to depend on temperature, especially those related to mass or charge transport, which could limit their applications, it being necessary to have studies that establish this dependence.

**Figure 2:**
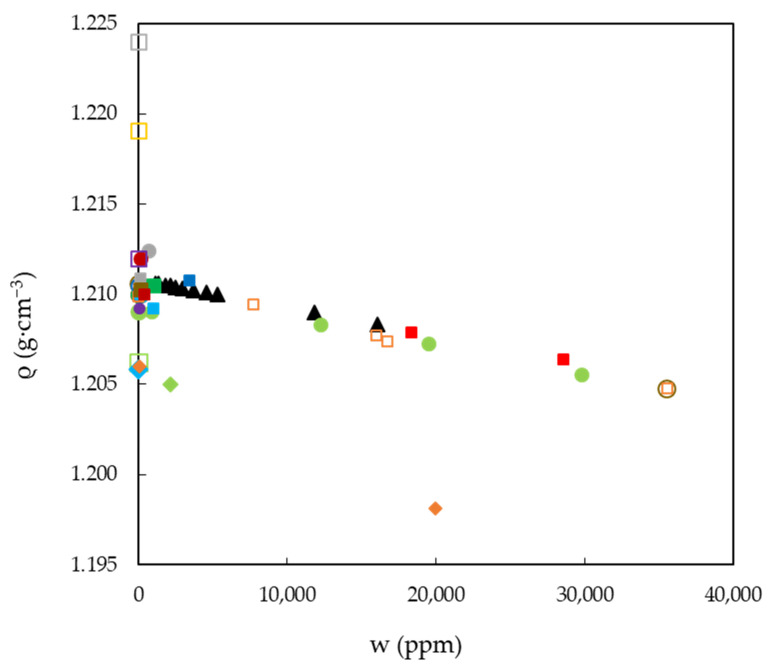
Experimental and published densities, ρ, of EAN as a function of the water content, w, at 25 °C. Symbols: present work (black solid triangles) and a compilation of those published data with an accurate measure of water content (solid) or not (open): [15] (red squares), [23] (green dots), [24] (yellow dots), [27] (orange dots), [28] (cyan dots), [29] (red dots), [32] (pink dots), [36] (grey dots), [41] (dark green dots), [46] (brown dots), [48] (blue dots), [52] (dark red dots), [58] (purple dots), [60] (green squares), [65] (yellow squares), [67] (orange squares), [70] (cyan squares), [71] (pink squares), [75] (grey squares), [76] (dark green squares), [77] (brown squares), [81] (blue squares), [82] (dark red squares), [85] (purple squares), [86] (green rhombs), [87] (cyan rhombs) and [89] (orange rhombs).

The aforementioned highlights the fact that, in order to properly design a process, the systematic and precise measurement of physicochemical properties is of the utmost importance. Despite this, we have found that the published results for a given property at the same temperature vary very significantly. According to all the data gathered in the literature, it could be assumed that water absorption from the atmosphere is one of the main reasons for the dispersion observed for a given property and temperature, since the two ILs studied here are hygroscopic substances, as are many ILs [90]. As a representative case, previously published densities (ρ) of EAN measured at 25 °C are presented in Figure 2, along with the experimental densities studied here. In this figure, the property as a function of the water content (w) accurately reported in the papers is represented using solid symbols, and with open ones if it was not. 

In view of the results, it seems critical to control and determine the water content if an accurate characterization is to be made. For this reason, in this work we have proposed as an objective to study systematically the influence of the content of small quantities of water (from about 300 to 30,000 ppm) in EAN and PAN on the measurement of density, viscosity and electrical conductivity from 5 to 95 °C, and the refractive index and surface tension at 25 °C. To our knowledge, this comprehensive study of EAN and PAN has not been carried out over such a wide temperature range with a strict control of water content. 

## 2. Results

**Figure 3:**
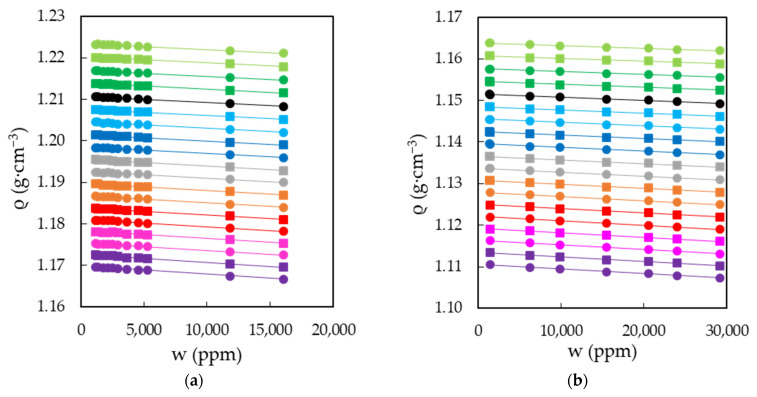
Densities, ρ, of EAN (**a**) and PAN (**b**) as a function of the water content, w, for the temperatures studied: 5 (green dots), 10 (green squares), 15 (dark green dots), 20 (dark green squares), 25 (black dots), 30 (cyan squares), 35 (cyan dots), 40 (blue squares), 45 (blue dots), 50 (grey squares), 55 (grey dots), 60 (orange squares), 65 (orange dots), 70 (red squares), 75 (red dots), 80 (pink squares), 85 (pink dots), 90 (purple squares) and 95 °C (purple dots). Solid lines were obtained from Equation (1).

**Figure 4:**
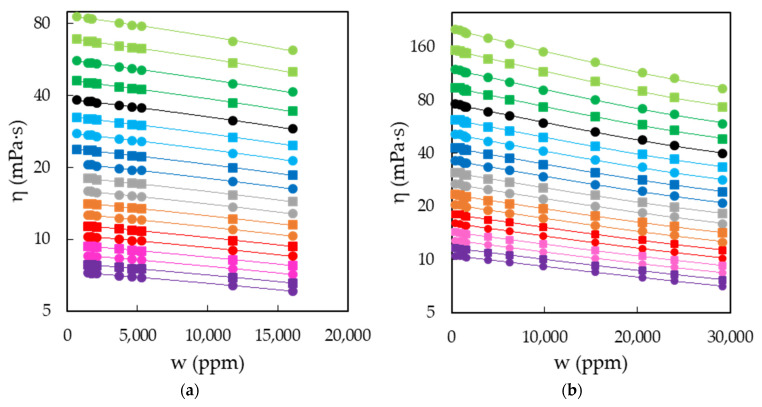
Viscosities, η, of EAN (**a**) and PAN (**b**) as a function of the water content, w, on a semi logarithmic scale for the temperatures studied: 5 (green dots), 10 (green squares), 15 (dark green dots), 20 (dark green squares), 25 (black dots), 30 (cyan squares), 35 (cyan dots), 40 (blue squares), 45 (blue dots), 50 (grey squares), 55 (grey dots), 60 (orange squares), 65 (orange dots), 70 (red squares), 75 (red dots), 80 (pink squares), 85 (pink dots), 90 (purple squares) and 95 °C (purple dots). Solid lines were obtained from Equation (1).

**Figure 5:**
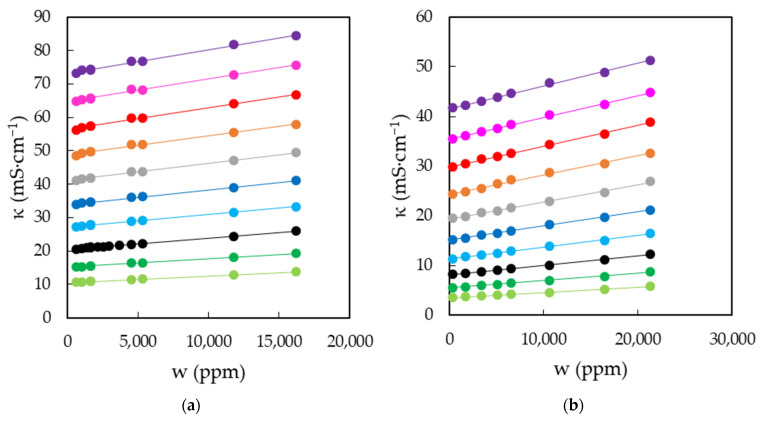
Electrical conductivities, κ, of EAN (**a**) and PAN (**b**) as a function of the water content, w, for the temperatures studied: 5 (green dots), 15 (dark green dots), 25 (black dots), 35 (cyan dots), 45 (blue dots), 55 (grey dots), 65 (orange dots), 75 (red dots), 85 (pink dots) and 95 °C (purple dots). Solid lines were obtained from Equation (1).

**Figure 6:**
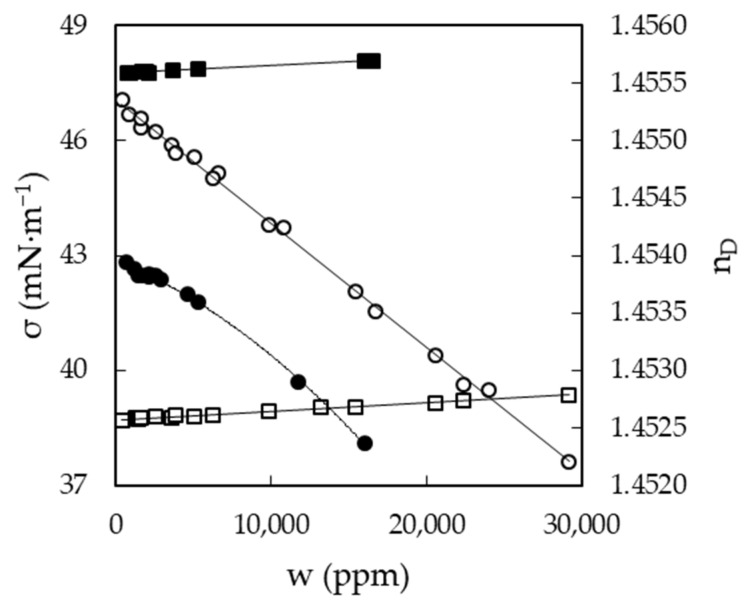
Refractive index, n_D_ (dots), and surface tension, σ (squares), of EAN (solid symbols) and PAN (open symbols) as a function of the water content, w, at 25 °C. Solid lines were obtained from Equation (1).

The characterization of EAN and PAN involves the measurement of physical properties covering a wide range of temperatures, between 5 and 95 °C, when possible. This was the case for density, viscosity and electrical conductivity, which were measured every 5 °C for the first two and 10 °C for the latter. Thus, Figure 3a,b shows the results obtained for the density of EAN and PAN as a function of the water content, respectively. In the same way, Figure 4a,b and Figure 5a,b display the experimental results of viscosity (on a semi logarithmic scale) and electrical conductivity. Finally, the refractive index and surface tension at 25 °C of both LIs are plotted in Figure 6. As can be seen, all the physical properties studied depend linearly on the composition in the concentration range studied, with the exception of the refractive index of EAN and the viscosity of PAN, whose relationship is better fitted through a polynomial of degree 2.

All the experimental results obtained are shown in Tables S1–S5 in the Supporting Materials section. In all cases, the data were fitted to an equation of the type:

(1) $$Q=Q_{\mathrm{IL}}+\sum_{i=1}^{N}A_{i}w^{i},$$

where Q is the property studied (ρ, η, κ, n_D_ or σ) of the IL at a given temperature and content in water (w), Q_IL_ is the corresponding property of the IL without any water content (ρ_IL_, η_IL_, κ_IL_, n_D,IL_ or σ_IL_) and Q_IL_ and A_i_ are fitting parameters. All the best values for the fitting parameters, Q_IL_ and A_i_, together with the coefficient of determinations, are shown in Table 1, Table 2, Table 3 and Table 4.

## 3. Discussion

### 3.1. Experimental Measurements as a Function of Water Content

Analyzing the results obtained from the perspective of the influence of a low water content on the physical characterization of EAN and PAN, it can be clearly observed that not all properties are equally affected. Thus, the quantities most significantly modified by the presence of water are those of transport. The percentage change in viscosity averaged over all temperatures between water-free content (η_IL_) and 30,000 ppm reaches −39% and −42% for EAN and PAN, respectively. Meanwhile, in the case of electrical conductivity, the corresponding percentage change is even greater, representing 43 and 56%, respectively. At the other extreme, we find refractive index (−0.11% and −0.22%, respectively), density (−0.42% and −0.22%) and surface tension (1.3% and 1.7%).

As can be deduced from the data obtained, the presence of small quantities of water generates a considerable increase in both the fluidity of the liquid and the mobility of the charge. Although from a volumetric and surface point of view the liquids do not undergo such a marked change, it can be sufficiently significant when a characterization of the liquid structure is pursued.

### 3.2. Experimental Measurements as a Function of Temperature

**Figure 7:**
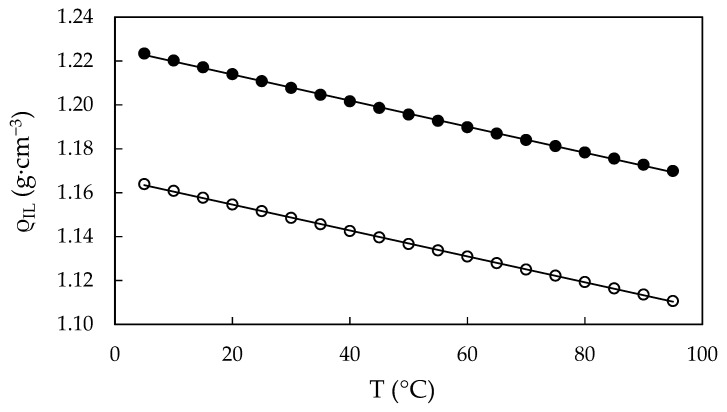
Calculated densities, ρ_IL_, of *water-free* EAN (solid dots) and PAN (open dots) as a function of the temperature, obtained from Equation (1). Solid line obtained from the best straight line.

**Figure 8:**
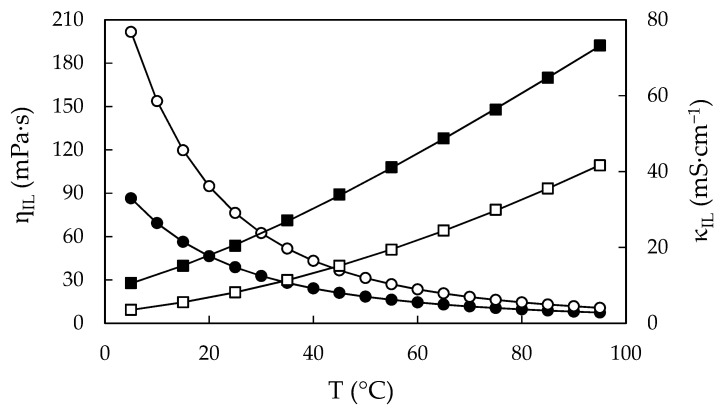
Calculated viscosities, η_IL_, and calculated electrical conductivities, κ_IL_, of *water-free* EAN (solid symbols) and PAN (open symbols) as a function of the temperature, obtained from Equation (1). Dots correspond to the left axis (viscosity) and squares to the right axis (electrical conductivity). Solid line obtained from VTF Equation (3).

From the fitting parameter *Q_IL_* of each property at each temperature, which corresponds to the values of the properties at water content w = 0, in Figure 7 and Figure 8 we have plotted the densities, viscosities and electrical conductivities of EAN and PAN as a function of temperature.

The density data follow a linear trend with temperature as follows:

(2) $$\rho_{\mathrm{IL}}=A_{\rho ,0}+A_{\rho ,1}T,$$

where ρ_IL_ is the density of water-free IL, T is the temperature in °C and A_ρ,0_ and A_ρ,1_ are the fitting parameters presented in Table 5. These densities for both *water-free* ILs present a very similar slope, differing by 1.3%.

In the case of viscosity and electrical conductivity, they can be correlated by the Vogel–Tammann–Fulcher (VTF) equation, as is usual for liquids of this nature [91]. This equation can be generally written as:

(3) $$Q_{\mathrm{IL}}=Q_{\infty }e^{B/\left(T+273,15-T_{0}\right)},$$

where Q_IL_ describes η_IL_ or κ_IL_, $Q_{\infty }$ is the limit of viscosity or electrical conductivity at infinite temperature, B is related to the activation energy of ions to flow or to the activation energy for the ion hopping and, finally, T_0_ is related with the glass transition temperature in K [12,92,93,94] and T is the temperature expressed in °C. The fitting parameters $Q_{\infty }$, B and T_0_ are given in Table 5, as well as the corresponding percentual deviation, which is defined as:

(4) $$s=100\;\sqrt{\frac{\sum_{i=1}^{N}{\left(\frac{Q_{\mathrm{IL},\;i}-Q_{\mathrm{VTF},i}}{Q_{\mathrm{IL},\;i}}\right)}^{2}}{N-1}},$$

where Q_VTF_ is the value of the calculated quantity obtained from the best fit of the VTF equation. The resulting curves are shown in Figure 8, showing a very good agreement with Q_IL_ data.

### 3.3. Experimental Measurements as a Function of Water Content and Temperature

The two properties most affected by the presence of water impurities can be expressed as a function of water content and temperature by means of a single equation. Thus, the fitting equation for the viscosity and electrical conductivity of the IL at a given water content and temperature is:

(5) $$Q\left(w,T\right)=Q_{\mathrm{IL}}\left(T\right)+\sum_{i=1}^{N}A_{Q,i}\left(T\right){\;w}^{i},$$

where Q(w,T) is η(w,T) or κ(w,T), Q_IL_(T) is the calculated water-free viscosity or electrical conductivity of the IL given by Equation (3) and the corresponding fitting parameters can be defined as:

(6) $$A_{\eta ,\;i\;}\left(T\right)={\left(-1\right)}^{i}e^{\overset{M}{\underset{j=0}{\sum }}C_{j}T^{j}},$$

(7) $$A_{\kappa ,i}\left(T\right)=\sum_{j=0}^{M}C_{j}T^{j},$$

T being the temperature in °C in the 5 to 95 °C range. Finally, parameter $C_{j}$ was the result of the best fit of parameter A_i_ from Equation (1) and is reported in Table 6 together with the coefficients of determination R^2^.

The values obtained from Equation (5) show a very good agreement with experimental data for all water contents and temperatures studied, resulting in findings very similar to those calculated from Equations (1)–(3). Thus, the average percentual deviation for viscosities of EAN and PAN are 0.4 and 1.0, respectively, while those for electrical conductivities are 0.7 and 1.2, respectively.

### 3.4. Comparison with Published Data

As mentioned in Introduction, an exhaustive review of the present state of the art in the densities, viscosities, electrical conductivities, refractive indexes and surface tension of EAN and PAN at different temperatures was performed. As a result, the published experimental physical properties here studied at 25 °C are represented in Figures S1–S10 along with the properties measured in this work. On the other hand, Table S5 is also included in this section, which compiles in detail the properties studied in each bibliographic reference. From this review, it can be deduced that:

- Previously published data show a wide dispersion in all the properties studied;
- A significant number of papers do not present accurate information on the water content of pure ILs. In general, these data are the ones that differ the most from those presented here (open symbols in Figures S1–S10);
- Overall, those papers that do report on water content are in good agreement with those presented here;
- The data presented here provide systematic measurements and homogeneity, as well as covering previously unpublished temperature or water content ranges.

## 4. Materials and Methods

### 4.1. Materials

Ethylammonium nitrate (EAN) and propylammonium nitrate (PAN) are room temperature yellow to orange liquids that are commercially available. Both ILs were supplied by Iolitec and their mass fraction purity and percentage of water content certified by the supplier were >0.97 and <2%, respectively. To preserve the ILs from moisture, the bottles were sealed with film and handled in a chamber in which the relative humidity was maintained at <15%.

### 4.2. Preparation of Materials

The preparation of the samples started with a drying process which was different depending on their water content. For contents higher than 1000 ppm, the water removal was carried out under a reduced pressure of 10 mbar at a temperature of 120 °C for 8 to 10 h. Thereafter, the samples of IL were prepared by weight by adding the required amount of water to cover a range of concentrations up to 30,000 ppm. For water contents below 1000 ppm, the IL was exposed to pressures of 10^−3^ mbar for 48 h and used without further modifications. At the end of both processes, the liquids were kept in a hermetically sealed flask with an internal argon atmosphere.

The final water contents (*w*) were determined using a Mettler Toledo coulometric Karl Fischer titrator C10S, the estimated uncertainty of which is 50 ppm.

### 4.3. Density and Viscosity

Densities (*ρ*) and viscosities (η) were determined using an Anton Paar Stabinger VTM 3000 viscodensimeter with a repeatability of 0.5 kg·m^−3^ and 0.4% of the measured value for viscosity. The device has an internal Peltier thermostat presenting an uncertainty of 0.02 °C in temperature.

### 4.4. Refractive Index

Refractive indexes (*n_D_*) were measured with an Anton Paar Abbemat-WR automatic refractometer with an uncertainty of 4 × 10^−5^. The device has an internal Peltier thermostat featuring an uncertainty of 0.03 °C.

### 4.5. Surface Tension

Surface tensions (*σ*) were determined using a Lauda TVT1 automated tensiometer, which presents an uncertainty of 0.02 mN·m^−1^. A Lauda RC6 CP thermostatic bath controlled the temperature with an uncertainty < 0.2 °C.

### 4.6. Electrical Conductivity

The electrical conductivity of the samples was measured by means of a Crison GLP 31 conductivity meter, which works with an alternating current of 500 Hz and a voltage of 4.5 V. The measuring cell Hach 52 92 connected to it operates in a range from 0.2 µS·cm^−1^ to 100 mS·cm^−1^. It also allows a working temperature range from −30 to 80 °C with a repeatability of 0.5%, it being possible to measure at higher temperatures as long as these are very stable. A Julabo F25 thermostatic bath controlled the temperature with an uncertainty of 0.1 °C.

## 5. Conclusions

This work contributes to an exhaustive physical characterization of EAN and PAN, two ILs widely used in many different fields of work. These are two hygroscopic liquids that require careful moisture control in their handling. In spite of the existence of a considerable number of articles published on their physical properties, it was found after a detailed bibliographic search that there is a great dispersion in the results obtained, and that in numerous cases, the water content of the pure materials was not provided.

In this work, several physical properties of EAN and PAN with water contents between 300 and 30,000 ppm were measured systematically. Thus, experimental densities, viscosities and electrical conductivity were reported for a wide range of temperatures between 5 and 95 °C, while the refractive index and surface tension were measured at 25 °C. As a result of this work, experimental measurements that have not previously been published over such a wide temperature range with a strict control of water content are provided.

The properties most significantly modified by the presence of water in EAN and PAN were those of transport. The calculated absolute value of the average percentage change from water-free to 30,000 ppm is between 39 and 56%. Finally, a correlation equation dependent on both water content in the IL and temperature was provided for viscosity and electrical conductivity, resulting in a very good fit to all experimental data.

## Figures and Tables

**Table 1:** Fitting Equation (1) to density data (g·cm^−3^) of EAN and PAN as a function of water content (ppm): parameters and coefficient of determination R^2^.

	EAN			PAN		
T (°C)	ρ_IL_	A_1_ (10^7^)	R^2^	ρ_IL_	A_1_ (10^7^)	R^2^
5	1.2234	−1.4448	0.9963	1.1639	−0.66515	0.9941
10	1.2202	−1.4610	0.9971	1.1608	−0.67382	0.9941
15	1.2171	−1.4745	0.9988	1.1577	−0.70588	0.9958
20	1.2140	−1.5136	0.9939	1.1546	−0.73063	0.9913
25	1.2108	−1.5354	0.9989	1.1516	−0.76511	0.9945
30	1.2077	−1.5516	0.9978	1.1485	−0.79664	0.9938
35	1.2046	−1.5800	0.9934	1.1456	−0.83059	0.9926
40	1.2016	−1.6151	0.9988	1.1425	−0.83760	0.9920
45	1.1986	−1.6151	0.9988	1.1396	−0.89210	0.9908
50	1.1956	−1.6805	0.9977	1.1366	−0.90778	0.9920
55	1.1927	−1.6700	0.9944	1.1337	−0.96657	0.9944
60	1.1898	−1.6959	0.9969	1.1309	−1.0122	0.9956
65	1.1869	−1.7551	0.9947	1.1279	−1.0033	0.9986
70	1.1840	−1.7654	0.9913	1.1250	−1.0411	0.9980
75	1.1812	−1.8331	0.9907	1.1222	−1.0732	0.9995
80	1.1783	−1.8467	0.9922	1.1193	−1.0732	0.9995
85	1.1755	−1.8688	0.9945	1.1164	−1.1194	0.9981
90	1.1727	−1.9341	0.9969	1.1136	−1.1428	0.9953
95	1.1699	−1.9799	0.9954	1.1106	−1.1107	0.9927

**Table 2:** Fitting Equation (1) to viscosity data (mPa·s) of EAN and PAN as a function of water content (ppm): parameters of Equation (1) and coefficient of determination R^2^.

	EAN			PAN			
T (°C)	η_IL_	A_1_ (10^4^)	R^2^	η_IL_	A_1_ (10^4^)	A_2_ (10^9^)	R^2^
5	86.5	−15.60	0.9981	201.6	−57.29	70.74	0.9990
10	69.3	−12.10	0.9983	153.7	−41.82	50.26	0.9990
15	56.3	−9.517	0.9983	119.7	−31.40	37.31	0.9989
20	46.3	−7.504	0.9984	94.8	−23.98	28.02	0.9989
25	38.7	−6.084	0.9982	76.3	−18.51	21.00	0.9988
30	32.7	−4.960	0.9989	62.3	−14.56	16.29	0.9988
35	27.9	−4.125	0.9985	51.6	−11.64	12.83	0.9987
40	24.05	−3.439	0.9987	43.2	−9.434	10.30	0.9984
45	20.92	−2.901	0.9987	36.5	−7.642	8.041	0.9987
50	18.33	−2.468	0.9987	31.2	−6.293	6.465	0.9986
55	16.19	−2.120	0.9989	26.9	−5.220	5.206	0.9983
60	14.40	−1.832	0.9989	23.39	−4.362	4.224	0.9982
65	12.88	−1.600	0.9989	20.56	−3.748	3.623	0.9989
70	11.59	−1.405	0.9990	18.15	−3.191	2.991	0.9991
75	10.48	−1.239	0.9988	16.11	−2.722	2.455	0.9993
80	9.53	−1.094	0.9987	14.40	−2.340	2.026	0.9995
85	8.69	−0.9741	0.9987	12.93	−2.021	1.673	0.9996
90	7.97	−0.8775	0.9989	11.68	−1.764	1.419	0.9998
95	7.34	−0.7932	0.9989	10.59	−1.534	1.157	0.9999

**Table 3:** Fitting Equation (1) to electrical conductivity data (mS·cm^−1^) of EAN and PAN as a function of water content (ppm): parameters and coefficient of determination R^2^.

	EAN			PAN		
T (°C)	κ_IL_	A_1_ (10^4^)	R^2^	κ_IL_	A_1_ (10^4^)	R^2^
5	10.55	1.989	0.9991	3.50	1.053	0.9982
15	15.10	2.566	0.9990	5.52	1.451	0.9987
25	20.4	3.436	0.9978	8.12	1.891	0.9986
35	27.1	3.849	0.9982	11.34	2.364	0.9984
45	33.9	4.469	0.9980	15.11	2.831	0.9990
55	41.1	5.141	0.9968	19.37	3.450	0.9954
65	48.7	5.780	0.9911	24.4	3.875	0.9927
75	56.3	6.504	0.9951	29.9	4.185	0.9984
85	64.7	6.795	0.9936	35.5	4.361	0.9981
95	73.2	7.087	0.9942	41.6	4.548	0.9974

**Table 4:** Fitting Equation (1) to refractive index and surface tension (mN·m^−1^) data at 25 °C of EAN and PAN as a function of water content (ppm): parameters and coefficient of determination R^2^.

	EAN				PAN		
Property	Q_IL_	A_1_	A_2_	R^2^	Q_IL_	A_1_	R^2^
n_D_	1.45395	−5.14135 × 10^−8^	−3.00678 × 10^−12^	0.9979	1.45536	−1.08059 × 10^−7^	0.9980
σ (mN·m^−1^)	47.75	2.008 × 10^−5^		0.9932	38.72	2.201 × 10^−5^	0.9929

**Table 5:** Fitting of density (g·cm^−3^), viscosity (mPa·s) and electrical conductivity (mS·cm^−1^) data of water-free EAN and PAN as function of temperature: parameters of Equations (2) and (3), coefficient of determination R^2^ and percentual deviation (s).

IL	A_ρ__,0_	A_ρ__,1_ (10^4^)	R^2^_ρ_	$Q_{\infty ,\;\eta }$	B_ƞ_	T_0,__ƞ_	s_ƞ_	$Q_{\infty ,\;\kappa }$	B_κ_	T_0,κ_	s_κ_
EAN	1.2257	−5.9379	0.9996	0.2111	778.8	148.7	0.06	666.2	−424.9	175.8	0.7
PAN	1.1664	−5.8998	0.9998	0.1823	869.5	154.1	0.10	904.1	−620.8	166.4	0.3

**Table 6:** Fitting of viscosity (mPa·s) and electrical conductivity (mS·cm^−1^) data of EAN and PAN as a function of water content and temperature: parameters of Equations (6) and (7) and coefficients of determination R^2^.

IL	EAN				PAN			
A (T)	C_0_	C_1_	C_2_	R^2^	C_0_	C_1_	C_2_	R^2^
A_κ_ (T)	1.554 × 10^−4^	7.445 × 10^−6^	−1.549 × 10^−8^	0.9954	6.294 × 10^−5^	5.924 × 10^−6^	−1.775 × 10^−8^	0.9923
A_ƞ__,1_ (T)	−6.246	−4.994 × 10^−2^	1.740 × 10^−4^	0.9997	−4.917	−5.883 × 10^−2^	1.951 × 10^−4^	0.9996
A_ƞ__,2_ (T)					−16.22	−6.074 × 10^−2^	1.633 × 10^−4^	0.9994

## Data Availability

Data is contained within the article or supplementary material.

## Supplementary Materials

The following are available online at https://www.mdpi.com/article/10.3390/ijms22147334/s1.
