# Supplementary material for: Influence of Small Quantities of Water on the Physical Properties of Alkylammonium Nitrate Ionic Liquids
Source: Int J Mol Sci. 2021 Jul 8;22(14):7334. doi: 10.3390/ijms22147334 (PMC8306069; doi:10.3390/ijms22147334)
Supplement: Supplementary file 1 [file ijms-22-07334-s001.zip › ijms-1273345-supplementary.pdf]

SUPPORTING MATERIALS

Table S1. Experimental densities (ρ) of EAN and PAN at different temperatures as function of water content (w).

Ionic liquid	w (ppm)	ρ (g·cm⁻³)																		
		T (°C)																		
		**5**	**10**	**15**	**20**	**25**	**30**	**35**	**40**	**45**	**50**	**55**	**60**	**65**	**70**	**75**	**80**	**85**	**90**	**95**
EAN	1140	1.2232	1.2201	1.2169	1.2138	1.2106	1.2076	1.2045	1.2014	1.1984	1.1955	1.1925	1.1896	1.1867	1.1838	1.1809	1.1781	1.1753	1.1725	1.1696
	1320	1.2233	1.2201	1.2169	1.2138	1.2106	1.2075	1.2045	1.2014	1.1984	1.1954	1.1925	1.1896	1.1865	1.1836	1.1808	1.1780	1.1752	1.1724	1.1697
	1760	1.2231	1.2199	1.2168	1.2136	1.2105	1.2074	1.2043	1.2013	1.1983	1.1953	1.1923	1.1894	1.1865	1.1836	1.1808	1.1779	1.1751	1.1723	1.1694
	2110				1.2137	1.2105	1.2074	1.2044	1.2013	1.1983	1.1953	1.1924	1.1894	1.1865	1.1836	1.1808	1.1779	1.1751	1.1723	1.1695
	2120	1.2231	1.2199	1.2167																
	2490	1.2231	1.2199	1.2167	1.2136	1.2104	1.2073	1.2042	1.2012	1.1982	1.1952	1.1923	1.1893	1.1865	1.1837	1.1809	1.1780	1.1752	1.1723	1.1694
	2930	1.2230	1.2198	1.2166	1.2134	1.2103	1.2072	1.2041	1.2011	1.1981	1.1951	1.1921	1.1892	1.1863	1.1834	1.1807	1.1779	1.1750	1.1722	1.1693
	3660	1.2229	1.2197	1.2165	1.2134	1.2102	1.2071	1.2040	1.2010	1.1980	1.1950	1.1920	1.1891	1.1862	1.1833	1.1804	1.1776	1.1748	1.1719	1.1691
	4580	1.2228	1.2196	1.2164	1.2133	1.2101	1.2070	1.2040	1.2009	1.1979	1.1949	1.1920	1.1890	1.1861	1.1832	1.1803	1.1775	1.1747	1.1718	1.1689
	5290	1.2227	1.2195	1.2163	1.2132	1.2100	1.2069	1.2038	1.2008	1.1978	1.1948	1.1918	1.1889	1.1860	1.1831	1.1802	1.1774	1.1746	1.1717	1.1689
	11790	1.2217	1.2185	1.2153	1.2122	1.2090	1.2059	1.2028	1.1997	1.1967	1.1937	1.1907	1.1878	1.1848	1.1819	1.1790	1.1762	1.1733	1.1704	1.1675
	16070	1.2211	1.2179	1.2147	1.2115	1.2083	1.2052	1.2021	1.1990	1.1960	1.1929	1.1900	1.1870	1.1840	1.1811	1.1782	1.1753	1.1725	1.1696	1.1667
PAN	1300	1.1639	1.1607	1.1576	1.1546	1.1515	1.1485	1.1455	1.1425	1.1396	1.1366	1.1337	1.1308							
	1430	1.1638	1.1607	1.1576	1.1545	1.1514	1.1484	1.1454	1.1424	1.1394	1.1365	1.1336	1.1307	1.1278	1.1249	1.1220	1.1191	1.1163	1.1135	1.1105
	6240	1.1635	1.1603	1.1572	1.1541	1.1511	1.1480	1.1450	1.1420	1.1390	1.1360	1.1331	1.1302	1.1273	1.1244	1.1215	1.1186	1.1157	1.1128	1.1098
	9890	1.1632	1.1601	1.1570	1.1538	1.1508	1.1477	1.1447	1.1416	1.1387	1.1357	1.1327	1.1298	1.1269	1.1240	1.1211	1.1182	1.1153	1.1124	1.1094
	15460	1.1628	1.1597	1.1565	1.1534	1.1503	1.1472	1.1442	1.1411	1.1381	1.1351	1.1322	1.1292	1.1263	1.1234	1.1205	1.1176	1.1147	1.1117	1.1087
	20580	1.1626	1.1595	1.1563	1.1532	1.1501	1.1470	1.1440	1.1409	1.1379	1.1349	1.1319	1.1289	1.1259	1.1230	1.1200	1.1171	1.1142	1.1113	1.1084
	23990	1.1623	1.1591	1.1560	1.1528	1.1497	1.1466	1.1435	1.1405	1.1374	1.1344	1.1314	1.1284	1.1255	1.1225	1.1196	1.1167	1.1138	1.1109	1.1079
	29190	1.1620	1.1588	1.1556	1.1525	1.1493	1.1462	1.1431	1.1401	1.1370	1.1340	1.1309	1.1279	1.1250	1.1220	1.1190	1.1161	1.1131	1.1102	1.1073

Table S2. Experimental viscosities (η) of EAN and PAN at different temperatures as function of water content (w).

Ionic liquid	w (ppm)	η (mPa·s)																		
		T (°C)																		
		**5**	**10**	**15**	**20**	**25**	**30**	**35**	**40**	**45**	**50**	**55**	**60**	**65**	**70**	**75**	**80**	**85**	**90**	**95**
EAN	660	85.7	68.8	55.9	46.0	38.4	32.4	27.7	23.85											
	1480	84.5	67.5	54.9	45.2	37.8	31.9	27.3	23.53	20.48	17.96	15.87	14.12	12.64	11.38	10.30	9.36	8.55	7.84	7.22
	1680	83.8	67.4	54.8	45.2	37.8	31.9	27.3	23.54	20.48	17.96	15.87	14.12	12.64	11.37	10.29	9.36	8.55	7.84	7.22
	1760	83.8	67.4	54.8	45.2	37.7	31.9	27.3	23.52	20.47	17.95	15.86	14.11	12.63	11.37	10.29	9.35	8.54	7.84	7.22
	2110		66.6	54.1	44.7	37.4	31.6	27.0	23.31	20.29	17.80	15.74	14.00	12.54	11.29	10.22	9.29	8.48	7.78	7.17
	3660	80.6	64.6	52.6	43.4	36.3	30.8	26.3	22.72	19.80	17.38	15.38	13.69	12.26	11.05	10.01	9.10	8.32	7.64	7.03
	4580	78.8	63.5	51.8	42.8	35.8	30.3	25.9	22.41	19.54	17.16	15.19	13.53	12.12	10.93	9.89	9.01	8.23	7.56	6.97
	5290	78.0	62.7	51.1	42.3	35.4	30.0	25.7	22.24	19.40	17.05	15.08	13.44	12.05	10.86	9.84	8.96	8.19	7.52	6.93
	11790	67.6	54.7	44.8	37.3	31.4	26.7	22.95	19.91	17.42	15.36	13.64	12.19	10.96	9.90	8.99	8.21	7.52	6.92	6.39
	16070	61.9	50.2	41.3	34.4	29.1	24.82	21.40	18.61	16.32	14.42	12.83	11.49	10.34	9.36	8.52	7.79	7.15	6.58	6.08
PAN	370	200.5	153.1	119.2	94.4	76.1	62.1	51.4	43.1	36.4	31.2	26.9	23.39	20.50	18.09	16.05	14.34	12.88	11.63	10.54
	870	198.6	151.5	118.2	93.8	75.5	61.7	51.1	42.9	36.3	31.0	26.8	23.29	20.41	18.01	15.99	14.28	12.81	11.55	10.46
	1300	194.5	148.6	115.8	91.9	74.1	60.5	50.1	41.9	35.4	30.3	26.0	22.56							
	1430	193.1	147.5	114.9	91.1	73.4	60.0	49.7	41.7	35.3	30.2	26.1	22.75	19.97	17.65	15.70	14.05	12.64	11.43	10.38
	1600	191.9	146.6	114.2	90.6	73.0	59.7	49.5	41.5	35.2	30.1	26.0	22.65	19.88	17.58	15.64	13.99	12.59	11.39	10.35
	3890	179.1	137.3	107.3	85.4	69.0	56.5	46.9	39.4	33.5	28.7	24.86	21.70	19.08	16.89	15.04	13.48	12.14	11.01	10.01
	6240	167.3	128.7	100.9	80.4	65.2	53.6	44.6	37.5	31.9	27.4	23.76	20.76	18.28	16.21	14.47	12.98	11.70	10.60	9.65
	9890	150.3	116.2	91.5	73.3	59.6	49.1	41.0	34.6	29.6	25.5	22.14	19.41	17.13	15.23	13.62	12.25	11.07	10.05	9.17
	15460	130.7	101.7	80.5	64.8	53.0	43.9	36.8	31.2	26.7	23.12	20.16	17.71	15.67	13.96	12.52	11.28	10.22	9.30	8.50
	20580	115.1	90.0	71.6	57.9	47.6	39.6	33.3	28.4	24.37	21.14	18.48	16.28	14.46	12.91	11.60	10.48	9.51	8.67	7.94
	23990	106.1	83.2	66.5	53.9	44.4	37.0	31.3	26.7	22.96	19.96	17.48	15.44	13.73	12.27	11.04	9.99	9.08	8.29	7.59
	29190	93.1	73.4	59.0	48.1	39.7	33.3	28.2	24.14	20.87	18.19	15.98	14.16	12.61	11.31	10.21	9.25	8.42	7.71	7.08

Table S3. Experimental electrical conductivities (κ) of EAN and PAN at different temperatures as function of water content (w).

Ionic liquid	w (ppm)	κ ($mS \cdot cm^{-1}$)									
		T (°C)									
		**5**	**15**	**25**	**35**	**45**	**55**	**65**	**75**	**85**	**95**
EAN	580	10.70	15.24	20.6	27.2	33.9	41.2	48.4	56.3	64.9	73.1
	1020	10.70	15.27	20.7	27.4	34.3	41.6	49.3	57.0	65.3	74.2
	1320			20.9							
	1620	10.92	15.57	21.0	27.7	34.7	41.9	49.8	57.3	65.7	74.4
	1640	10.86	15.52	21.1	27.9	34.6	41.9	49.6	57.5	65.6	74.1
	2120			21.2							
	2530			21.3							
	2930			21.4							
	3660			21.7							
	4540	11.44	16.30	22.0	28.9	36.0	43.8	51.8	59.8	68.5	76.8
	5310	11.64	16.49	22.2	29.2	36.3	43.8	51.8	59.8	68.1	76.8
	11750	12.87	18.08	24.3	31.6	39.0	47.0	55.4	64.0	72.7	81.7
	16170	13.78	19.25	26.1	33.3	41.1	49.4	57.9	66.7	75.5	84.4
PAN	310	3.54	5.58	8.19	11.39	15.18	19.55	24.4	29.9	35.5	41.7
	1630	3.69	5.77	8.42	11.76	15.54	19.93	24.8	30.5	36.1	42.2
	3350	3.86	6.02	8.78	12.18	16.11	20.6	25.5	31.4	37.0	43.0
	5050	4.01	6.23	9.04	12.51	16.52	21.0	26.5	32.0	37.6	43.9
	6540	4.19	6.48	9.38	12.87	16.99	21.7	27.3	32.6	38.4	44.7
	10570	4.59	7.05	10.11	13.90	18.20	23.0	28.8	34.4	40.3	46.7
	16430	5.17	7.83	11.12	15.09	19.64	24.7	30.5	36.5	42.4	48.8
	21340	5.80	8.68	12.23	16.47	21.2	27.0	32.6	38.9	44.8	51.3

Table S4. Experimental refractive indexes (n_D) and surface tensions (σ) of EAN and PAN at 25 °C as function of water content (w).

**Ionic liquid**	**w (ppm)**	**n_D**	**w (ppm)**	**σ (mN·m⁻¹)**
		**25 °C**		
EAN	660	1.45394	740	47.76
	1140	1.45388	900	47.77
	1480	1.45383	1680	47.79
	1740	1.45383	1740	47.80
	2110	1.45381	1760	47.79
	2120	1.45384	1940	47.79
	2530	1.45382	2110	47.78
	2930	1.45380	3660	47.83
	4580	1.45367	5290	47.87
	5290	1.45360	16070	48.08
	11790	1.45290	16550	48.08
	16070	1.45236		
PAN	370	1.45535	370	38.70
	870	1.45523	1300	38.76
	1600	1.45519	1430	38.73
	1620	1.45511	1600	38.77
	2560	1.45508	1620	38.77
	3560	1.45500	2560	38.79
	3890	1.45489	3560	38.78
	5080	1.45486	3890	38.82
	6240	1.45468	5080	38.81
	6550	1.45472	6240	38.84
	9890	1.45426	9890	38.93
	10820	1.45425	13230	39.03
	15460	1.45368	15460	39.04
	16730	1.45351	20580	39.16
	20580	1.45313	22370	39.22
	22370	1.45288	29190	39.36
	23990	1.45283		
	29190	1.45220		

Table S5 compiles in detail the properties studied at any temperature in each bibliographic reference. From this review, it can be deduced that previously published data show a wide dispersion in all the properties studied, and that a significant number of papers do not present accurate information on the water content of pure ILs.

Table S5. Compilation of published experimental densities (ρ), viscosities (η), electrical conductivities (κ), refractive indexes (n_D) and surface tensions (σ) of EAN and PAN.

REFERENCES	EAN	PAN
S1. Allen, M.; Evans, D. F.; Lumry, R. Thermodynamic properties of the ethylammonium nitrate + water system: Partial molar volumes, heat capacities, and expansivities. *J. Sol. Chem.* **1985**, *14*, 549-560.	ρ	
S2. Anaredy, R. S.; Lucio, A. J.; Shaw, S. K. Adventitious Water Sorption in a Hydrophilic and a Hydrophobic Ionic Liquid: Analysis and Implications. *ACS Omega* **2016**, *1*, 407−416.	ρ η	
S3. Angell, C.A.; Xu, W.; Belieres, J.; Yoshizawa, M. Ionic liquids and ionic liquid acids with high temperature stability for fuel cell and other high temperature applications, method of making and cell employing same. US Pantent US 7,867,658 B2, filed 3 May 2004 and issued 11 January 2011	ρ η κ	
S4. Anouti, M.; Caillon-Caravanier, M.; Dridi, Y.; Galiano, H.; Lemordant, D. Synthesis and Characterization of New Pyrrolidinium Based Protic Ionic Liquids. Good and Superionic Liquids. *J. Phys. Chem. B* **2008**, *112*, 13335–13343.	κ	
S5. Atkin, R.; Warr, G. G. Structure in Confined Room-Temperature Ionic Liquids. *J. Phys. Chem. C* **2007**, *111*, 5162-5168.	ρ	ρ
S6. Atkin, R.; Warr, G. G. The Smallest Amphiphiles: Nanostructure in Protic Room-Temperature Ionic Liquids with Short Alkyl Groups. *J. Phys. Chem. B* **2008**, *112*, 4164-4166.	ρ	ρ
S7. Atkin, R.; Warr, G. G. Bulk and Interfacial Nanostructure in Protic Room Temperature Ionic Liquids. In Ionic Liquids: From Knowledge to Application; Plechkova, N. V., Rogers, R. D., Seddon, K. R.; *American Chemical Society*: Washington DC, USA, **2009**; *ACS Symposium Series Volume 1030*, pp. 317-333.	ρ	ρ
S8. Barycki, M.; Sosnowska, A.; Gajewicz, A.; Bobrowski, M.; Wileńska, D.; Skurski, P.; Gieldoń, A.; Czaplewski, C.; Uhl, S.; Laux, E.; Journot, T.; Jeandupeux, L.; Keppner, H.; Puzyn, T. Temperature-dependent structure-property modeling of viscosity for ionic liquids. *Fluid Phase Equilib.* **2016**, *427*, 9- 17.	ρ η	
S9. Belieres, J.; Gervasio, D.; Angell, A. Binary inorganic salt mixtures as high conductivity liquid electrolytes for >100 °C fuel cells. *Chem. Commun.* **2006**, 4799–4801.	κ	
S10. Benhlima, N.; Turmine, M.; Letellier, P.; Naejus, R.; Lemordant, D. Étude électrochimique du nitrate d'éthylammonium fondu à 298 K : établissement d'une echelle de potentiel redox. *J. Chim. Phys.* **1998**, *95*, 25-44.	ρ η κ σ	

S11. Berthod, A; Ruiz-Ángel, M. J.; Carda-Broch, S. Ionic liquids in separation techniques. *J. Chromatogr. A* **2008**, *1184*, 6-18.	ρ η n_D	ρ η n_D
S12. Bonetti, M.; Oleinikova, A.; Bervillier, C. Coexistence Curve of the Ionic Binary Mixture Ethylammonium Nitrate-n-Octanol: Critical Properties. *J. Phys. Chem. B* **1997**, *101*, 2164-2173.	n_D	
S13. Campetella, M.; Mariani, A.; Sadun, C.; Wu, E.; Castner, E. W.; Gontrani, L. Structure and dynamics of propylammonium nitrate-acetonitrile mixtures: An intricate multi-scale system probed with experimental and theoretical techniques. *J. Chem. Phys.* **2018**, *148*, 134507.		η
S14. Canongia Lopes, J. N.; Esperança, J. M. S. S.; Mão de Ferro, A.; Pereiro, A. B.; Plechkova, N . V.; Rebelo, L. P. N.; Seddon, K. R.; Vázquez-Fernández, I. Protonic Ammonium Nitrate Ionic Liquids and Their Mixtures: Insights into Their Thermophysical Behavior. *J. Phys. Chem. B* **2016**, *120*, 2397–2406.	ρ η κ	ρ η κ
S15. Capelo, S. B.; Méndez-Morales, T.; Carrete, J.; López Lago, E.; Vila, J.; Cabeza, O.; Rodríguez, J. R.; Turmine, M.; Varela, L. M. Effect of Temperature and Cationic Chain Length on the Physical Properties of Ammonium Nitrate-Based Protic Ionic Liquids. *J. Phys. Chem. B* **2012**, *116*, 11302-11312.	ρ η κ σ n_D	ρ η κ σ n_D
S16. Chagnes, A.; Tougui, A.; Carré, B.; Ranganathan, N.; Lemordant, D. Abnormal Temperature Dependence of the Viscosity of Ethylammonium Nitrate–Methanol Ionic Mixtures. *J. Sol. Chem.* **2004**, *33*, 247-255.	η	
S17. Evans, D. F.; Yamauchi, A.; Roman, R.; Casassa, E. Z. Micelle Formation in Ethylammonium Nitrate, a Low-Melting Fused Salt. *J. Colloid Interface Sci.* **1982**, *88*, 89-96.	η σ	
S18. Evans, D. F.; Yamauchi, A.; Wei, G. J.; Bloomfleid, V. A. Micelle Size in Ethylammonium Nitrate As Determined by Classical and Quasi-Elastic Light Scattering. *J. Phys. Chem.* **1983**, *87*, 3537–3541.	η σ n_D	
S19. Frost, D. S.; Ngan, M.; Dai, L. L. Spontaneous Transport of Microparticles across Liquid−Liquid Interfaces. *Langmuir* **2013**, *29*, 9310–9315.	ρ η	
S20. Frost, D. S.; Machas, M.; Perea, B.; Dai, L. L. Nonconvective Mixing of Miscible Ionic Liquids. *Langmuir* **2013**, *29*, 10159−10165.	η n_D	
S21. Greaves, T. L.; Weerawardena, A.; Fong, C.; Krodkiewska, I.; Drummond, C. J. Protic Ionic Liquids: Solvents with Tunable Phase Behavior and Physicochemical Properties. *J. Phys. Chem. B* **2006**, *110*, 22479-22487.	ρ η κ σ n_D	
S22. Greaves, T. L.; Weerawardena, A.; Fong, C.; Drummond, C. J. Formation of Amphiphile Self-Assembly Phases in Protic Ionic Liquids. *J. Phys. Chem. B* **2007**, *111*, 4082-4088.	η σ	
S23. Greaves, T. L.; Drummond, J. Protic Ionic Liquids: Properties and Applications. *Chem. Rev.* **2008**, *108*, 206-237.	ρ η σ n_D	ρ η σ n_D
S24. Greaves, T. L.; Mudie, S. T.; Drummond, C. J. Effect of protic ionic liquids (PILs) on the formation of non-ionic dodecyl poly(ethylene	σ	

oxide) surfactant self-assembly structures and the effect of these surfactants on the nanostructure of PILs. *Phys. Chem. Chem. Phys.* **2011**, *13*, 20441-20452.		
S25. Hadded, M.; Biquard, M.; Letellier, P.; Schaal, R. Proprietes volumiques du nitrate d'ethylammonium fondu a 298 K et de ses melanges avec l'eau. *Can. J. Chem.* **1985**, *63*, 565-570.	ρ	
S26. Hadded, M.; Bahri, H.; Letellier, P. Tensions superficielles des mélanges binaires EAU-Nitrate d'éthylammonium à 298 K. *J. Chim. Phys.* **1986**, *83*, 419-426.	σ	
S27. Hadded, M.; Mayaffre, A.; Letellier, P.; Tensions superficielles des Sol.s idéales: Application aux solvants binaires constitués de methanol et de nitrate d'ethylammonium fondu a 298 K. *J. Chim. Phys.* **1989**, *86*, 525-537.	ρ σ	
S28. Hjalmarsson, N.; Atkin, R.; Rutland, M. W. Effect of Lithium Ions on Rheology and Interfacial Forces in Ethylammonium Nitrate and Ethanolammonium Nitrate. *J. Phys. Chem. C* **2016**, *120*, 26960–26967.	η	
S29. Hjalmarsson, N.; Atkin, R.; Rutland, M. W. Is the boundary layer of an ionic liquid equally lubricating at higher temperature?. *Phys. Chem. Chem. Phys.* **2016**, *18*, 9232-9239.	η	
S30. Kaneko, K.; Yoshimura, Y.; Shimizu, A. Water concentration dependence of the refractive index of various ionic liquid-water mixtures. *J. Mol. Liq.* **2018**, *250*, 283- 286.	n_D	n_D
S31. Kanzaki., R.;Uchida, K.; Song, X.; Umebayashi, Y.; Ishiguro, S. Acidity and Basicity of Aqueous Mixtures of a Protic Ionic Liquid, Ethylammonium Nitrate. *Anal. Sci.* **2008**, *24*, 1347-1349.	ρ	
S32. Krueger, M.; Bründermann, E.; Funkner, S.; Weingärtner, H.; Havenith, M. Communications: Polarity fluctuations of the protic ionic liquid ethylammonium nitrate in the terahertz regime. *J. Chem. Phys.* **2010**, *132*, 101101.	κ	
S33. Kundu, N.; Roy, A.; Dutta, R.; Sarkar, N. Translational and Rotational Diffusion of Two Differently Charged Solutes in Ethylammonium Nitrate–Methanol Mixture: Does the Nanostructure of the Amphiphiles Influence the Motion of the Solute?. *J. Phys. Chem. B* **2016**, *120*, 5481–5490.	η	
S34. López-Barrón, C. R.; Wagner, N. J. Structural Transitions of CTAB Micelles in a Protic Ionic Liquid. *Langmuir* **2012**, *28*, 12722–12730.	ρ η κ	
S35. Malham, I. B.; Letellier, P.; Mayaffre, A.; Turmine, M. Part I: Thermodynamic analysis of volumetric properties of concentrated aqueous Sol.s of 1-butyl-3-methylimidazolium tetrafluoro borate, 1-butyl-2,3-dimethylimidazolium tetrafluoroborate, and ethylammonium nitrate based on pseudo-lattice theory. *J. Chem. Thermodyn.* **2007**, *39*, 1132-1143.	ρ	
S36. Mariani, A.; Russina, O., Caminiti, R.; Triolo, A. Structural organization in a methanol:ethylammonium nitrate (1 :4) mixture: A joint X-ray /Neutron diffraction and computational study. *J. Mol. Liq.* **2015**, *212*, 947-956.	ρ	

S37. Mariani, A.; Caminiti, R.; Ramondo, F.; Salvitti, G.; Mocci, F.; Gontrani, L. Inhomogeneity in Ethylammonium Nitrate–Acetonitrile Binary Mixtures: The Highest "Low q Excess" Reported to Date. *J. Phys. Chem. Lett.* **2017**, *8*, 3512–3522.	ρ	
S38. Mariani, A.; Bonomo, M.; Wu, B.; Centrella, B.; Dini, D.; Castner Jr., E. W.; Gontrani, L. Intriguing transport dynamics of ethylammonium nitrate–acetonitrile binary mixtures arising from nano-inhomogeneity. *Phys. Chem. Chem. Phys.* **2017**, *19*, 27212—27220.	η κ	
S39. Méndez-Morales, T.; Carrete, J.; Cabeza, O.; Russina, O.; Triolo, A.; Gallego, L. J.; and Varela, L. M. Solvation of Lithium Salts in Protic Ionic Liquids: A Molecular Dynamics Study. *J. Phys. Chem. B* **2014**, *118*, 761–770.	ρ	
S40. Moore, L. J.; Summers, M. D.; Ritchie, A. D. Optical trapping and spectroscopic characterisation of ionic liquid Sol.s. *Phys. Chem. Chem. Phys.* **2013**, *15*, 13489-13498.	n_D	
S41. Oleinikova, A.; Bonetti, M. Critical Behavior of the Electrical Conductivity of Concentrated Electrolytes: Ethylammonium Nitrate in n-Octanol Binary Mixture. *J. Sol. Chem.* **2002**, *31*, 397-413.	η κ	
S42. Perlt, E.; Ray, P.; Hansen, A.; Malberg, F.; Grimme, S.; Kirchner, B. Finding the best density functional approximation to describe interaction energies and structures of ionic liquids in molecular dynamics studies. *J. Chem. Phys.* **2018**, *148*, 193835.	ρ	
S43. Perron, G.; Hardy, A.; Justice, J.-C.; Desnoyers, J. E. Model System for Concentrated Electrolyte Sol.s: Thermodynamic and Transport Properties of Ethylammonium Nitrate in Acetonitrile and in Water. *J. Sol. Chem.* **1993**, *22*, 1159-1178.	ρ η κ	
S44. Poole, C. F.; Kersten, B. R.; Ho, S. S.; Coddens, M. E.; Furton, K. G. Organic Salts, Liquid at Room Temperature, as Mobile Phases in Liquid Chromatography. *J. Chromatogr. A* **1986**, *352*, 407–425.	ρ η n_D	ρ η n_D
S45. Poole, C. F. Chromatographic and spectroscopic 1nethods f or the determination of solvent properties of room temperature ionic liquids. *J. Chromatogr. A* **2004**, *1037*, 49–82.	ρ η n_D	ρ η n_D
S46. Porcedda, S.; Marongiu, B.; Schirru, M.; Falconieri, D.; Piras, A. Excess enthalpy and excess volume for binary systems of two ionic liquids + water. *J. Therm. Anal. Calorim.* **2011**, *103*, 29–33.	ρ	ρ
S47. Prabhu, S. R.; Dutt, G. B. Does Addition of an Electrolyte Influence the Rotational Diffusion of Nondipolar Solutes in a Protic Ionic Liquid?. *J. Phys. Chem. B* **2015**, *119*, 6311–6316.	η	
S48. Ridings, C.; Warr, G. G.; Andersson, G. G. Surface Ordering in Binary Mixtures of Protic Ionic Liquids. *J. Phys. Chem. Lett.* **2017**, *8*, 4264–4267.	σ	σ
S49. Russina, O.; Mariani, A.; Caminiti, R.; Triolo, A. Structure of a Binary Mixture of Ethylammonium Nitrate and Methanol. *J. Sol. Chem.* **2015**, *44*, 669–685.	ρ	
S50. Segade, L.; Cabanas, M.; Domínguez-Pérez, M.; Rilo, E.; García-Garabal, S.; Turmine, M.; Varela, L. M.; Gómez-González, V.; Docampo-	ρ σ n_D	ρ σ n_D

Álvarez, B.; Cabeza, O. Surface and bulk characterisation of mixtures containing alkylammonium nitrates and water or ethanol: Experimental and simulated properties at 298.15 K. *J. Mol. Liq.* **2016**, *222*, 663-670.		
S51. Shetty, P. H.; Youngberg, P. J.; Kersen, B. R.; Poole, C. F. Solvent properties of liquid organic salts used as mobile phases in microcolumn reversed-phase liquid chromatography. *J. Chromatogr. A* **1987**, *411*, 61-79.	ρ η n_D	ρ η n_D
S52. Shotwell, J. B.; Flowers II, R. A. Electrochemical Investigation of the Solvolytic Properties of Ethylammonium Nitrate (EAN) and Propylammonium Nitrate (PAN). *Electroanalysis* **2000**, *12*, 223-226.	η	η
S53. Smith, J. A.; Webber, G. B.; Warr, G. G.; Atkin, R. Rheology of Protic Ionic Liquids and Their Mixtures. *J. Phys. Chem. B* **2013**, *117*, 13930–13935.	ρ η n_D	ρ η n_D
S54. Smith, J. A.; Webber, G. B.; Warr, G. G.; Zimmer, A.; Atkin, R.; Werzer, O. Shear dependent viscosity of poly(ethylene oxide) in two protic ionic liquids. *J. Colloid Interface Sci.* **2014**, *430*, 56-60.	ρ η	ρ η
S55. Song, X.; Hamano, H.; Minofar, B.; Kanzaki, R.; Fujii, K.; Kameda, Y.; Kohara, S.; Watanabe, M.; Ishiguro, S.; Umebayashi, Y. Structural Heterogeneity and Unique Distorted Hydrogen Bonding in Primary Ammonium Nitrate Ionic Liquids Studied by High-Energy X-ray Diffraction Experiments and MD Simulations. *J. Phys. Chem. B* **2012**, *116*, 2801–2813.	ρ	ρ
S56. Sonnleitner, T.; Nikitina, V.; Nazet, A.; Buchner, R. Do H-bonds explain strong ion aggregation in ethylammonium nitrate + acetonitrile mixtures?. *Phys. Chem. Chem. Phys.* **2013**, *15*, 18445-18452.	ρ η κ	
S57. Sonnleitner, T.; Turton, D. A.; Hefter, G.; Ortner, A.; Waselikowski, S.; Walther, M.; Wynne, K.; Buchner, R. Ultra-Broadband Dielectric and Optical Kerr-Effect Study of the Ionic Liquids Ethyl and Propylammonium Nitrate. *J. Phys. Chem. B* **2015**, *119*, 8826–8841.	ρ η κ	ρ η
S58. Sugden, S.; Wilkins, H. CLXVII.—The parachor and chemical constitution. Part XII. Fused metals and salts. *J. Chem. Soc.* **1929**, 1291-1298.	ρ σ	
S59.Thater, J. C.; Gérard, V.; Stubenrauch, C. Microemulsions with the Ionic Liquid Ethylammonium Nitrate: Phase Behavior, Composition, and Microstructure. *Langmuir* **2014**, *30*, 8283–8289.	ρ	
S60. Turton, D. A.; Sonnleitner, T.; Ortner, A.; Walther, M.; Hefter, G.; Seddon, K. R.; Stana, S.; Plechkova, N. V.; Buchner, R.; Wynne, K. Structure and dynamics in protic ionic liquids: A combined optical Kerr-effect and dielectric relaxation spectroscopy study. *Faraday Discuss.* **2012**, *154*, 145–153.	η	
S61. Usula, M.; Matteoli, E.; Leonelli, F.; Mocci, F.; Marincola, F. C.; Gontrani, L.; Porcedda, S. Thermo-physical properties of ammonium-based ionic liquid + N-methyl-2-pyrrolidone mixtures at 298.15 K. *Fluid Phase Equilib.* **2014**, *383*, 49-54.	ρ	ρ

S62. Wakeham, D.; Nelson, A.; Warr, G. G., Atkin, R. Probing the protic ionic liquid surface using X-ray reflectivity. *Phys. Chem. Chem. Phys.* **2011**, *13*, 20828–20835.	ρ	ρ
S63. Wakeham, D.; Eschebach, D.; Webber, G. B.; Atkin, R.; Warr, G. G. Surface Composition of Mixtures of Ethylammonium Nitrate, Ethanolammonium Nitrate, and Water. *Aust. J. Chem.* **2012**, *65*, 1554–1556.	σ	
S64. Weingärtner, H.; Merkel, T.; Käshammer, S.; Schröer, W.; Wiegand, S. The Effect of Short-Range Hydrogen-Bonded Interactions on the Nature of the Critical Point of Ionic Fluids. Part I: General Properties of the New System Ethylammonium Nitrate+n-Octanol with an Upper Consolute Point Near Room Temperature. *Ber. Bunsenges. Phys. Chem.* **1993**, *97*, 970-975.	κ	
S65. Weingärtner, H.; Knocks, A.; Schrader, W.; Kaatze, U. Dielectric Spectroscopy of the Room Temperature Molten Salt Ethylammonium Nitrate. *J. Phys. Chem. A* **2001**, *105*, 8646-8650.	ρ η κ n_D	
S66. Yalcin, D.; Drummond, C. J.; Greaves, T. L. High throughput approach to investigating ternary solvents of aqueous non-stoichiometric protic ionic liquids. *Phys. Chem. Chem. Phys.* **2019**, *21*, 6810-6827.	ρ σ	
S67. Zarrougui, R.; Dhahbi, M.; Lemordant, D. Volumetric Properties of Ethylammonium Nitrate + γ -Butyrolactone Binary Systems: Solvation Phenomena from Density and Raman Spectroscopy. *J. Sol. Chem.* **2010**, *39*, 1531–1548.	ρ	
S68. Zarrougui, R.; Dhahbi, M.; Lemordan, D. Electrochemical behaviour of iodine redox couples in aprotic and protic RTILs: 1-Butyl-1-methylpyrrolidinium bis(trifluoromethanesulfonyl)imide and ethylammonium nitrate. *J. Electroanal. Chem.* **2014**, *717-718*, 189 – 195.	η κ	
S69. Zarrougui, R.; Dhahbi, M.; Lemordant, D. Transport and Thermodynamic Properties of Ethylammonium Nitrate–Water Binary Mixtures: Effect of Temperature and Composition. *J. Sol. Chem.* **2015**, *44*, 686-702.	ρ η κ	

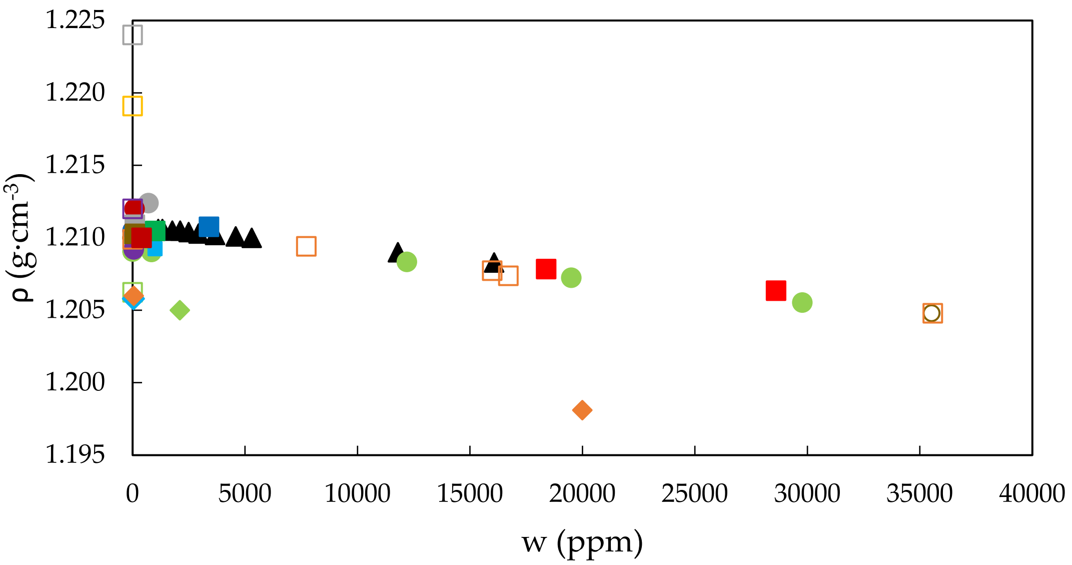

Figure S1. Density experimental data (ρ) of EAN at 25 °C of the present work (black solid triangles) and a compilation of that published near water content studied (w), with accurate measure of water content (solid) or not (open): [S1] (green dots), [S2] (yellow dots), [S5] (orange dots), [S6] (cyan dots), [S7] (red dots), [S10] (pink dots), [S14] (grey dots), [S19] (dark green dots), [S25] (brown dots), [S27] (blue dots), [S31] (dark red dots), [S37] (purple dots), [S39] (green squares), [S44] (yellow squares), [S46] (orange squares), [S49] (cyan squares), [S50] (red squares), [S51] (pink squares), [S55] (grey squares), [S56] (dark green squares), [S57] (brown squares), [S61] (blue squares), [S62] (dark red squares), [S65] (purple squares), [S66] (green rombs), [S67] (cyan rombs) and [S69] (orange rombs).

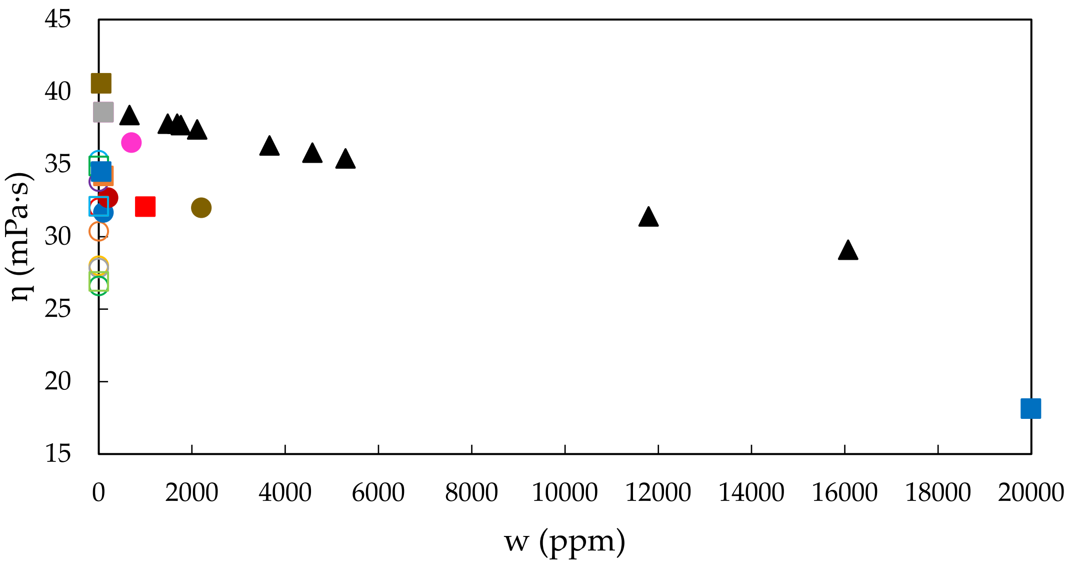

Figure S2. Viscosity experimental data (η) of EAN at 25 °C of the present work (black solid triangles) and a compilation of that published near water content studied (w), with accurate measure of water content (solid) or not (open): [S2] (green dots), [S3] (yellow dots), [S8] (orange dots), [S10] (cyan dots), [S11] (red dots), [S14] (pink dots), [S15] (grey dots), [S18] (dark green dots), [S22] (brown dots), [S33] (blue dots), [S38] (dark red dots), [S43] (purple dots), [S44] (green squares), [S45] (yellow squares), [S47] (orange squares), [S51] (cyan squares), [S53] (red squares), [S56] (pink squares), [S57] (grey squares), [S65] (dark green squares), [S68] (brown squares) and [S69] (blue squares).

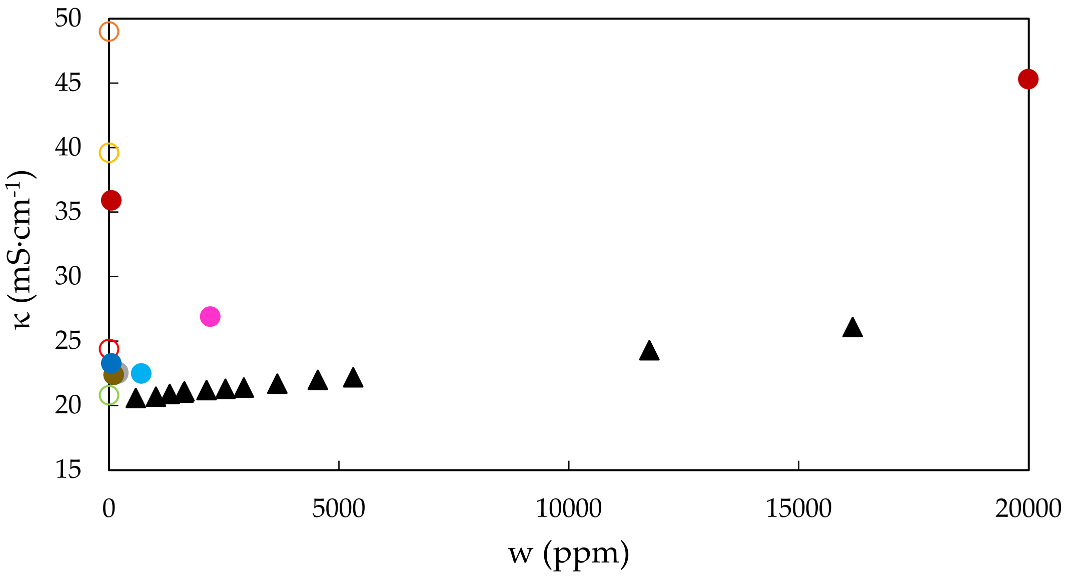

Figure S3. Electrical conductivity experimental data (κ) of EAN at 25 °C of the present work (black solid triangles) and a compilation of that published near water content studied (w), with accurate measure of water content (solid) or not (open): [S3] (green dots), [S4] (yellow dots), [S10] (orange dots), [S14] (cyan dots), [S15] (red dots), [S21] (pink dots), [S38] (grey dots), [S56] (dark green dots), [S57] (brown dots), [S68] (blue dots) and [S69] (dark red dots).

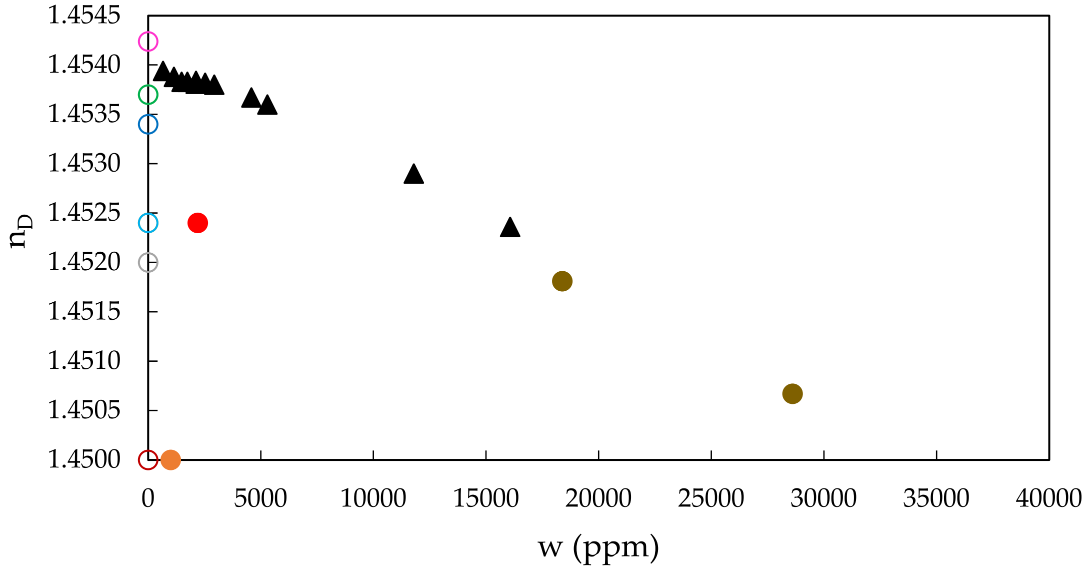

Figure S4. Refractive index experimental data (n_D) of EAN at 25 °C of the present work (black solid triangles) and a compilation of that published near water content studied (w), with accurate measure of water content (solid) or not (open): [S11] (green dots), [S15] (yellow dots), [S18] (orange dots), [S20] (cyan dots), [S21] (red dots), [S40] (pink dots), [S44] (grey dots), [S45] (dark green dots), [S50] (brown dots), [S51] (blue dots) and [S65] (dark red dots).

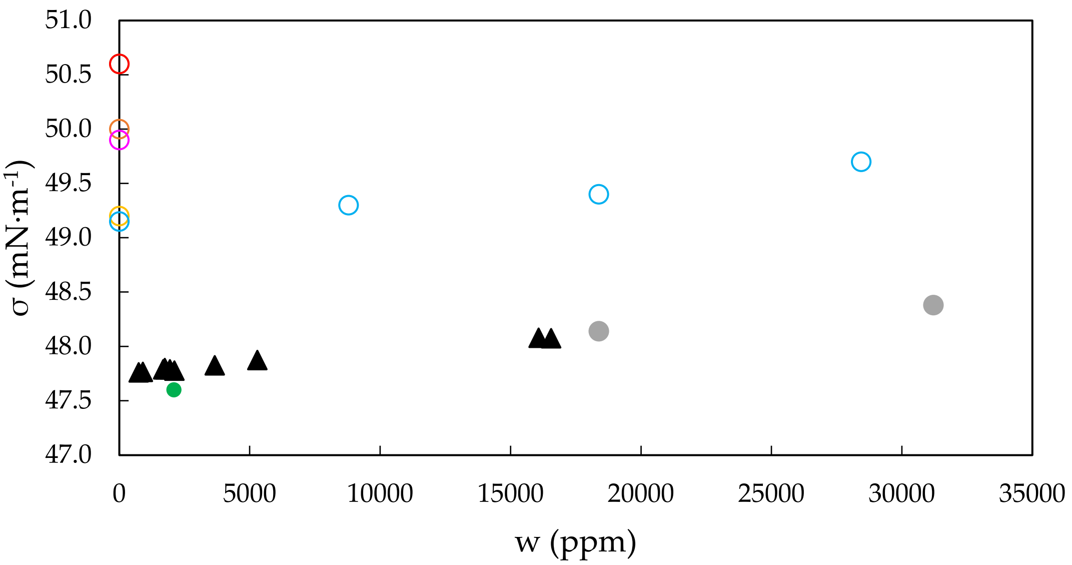

Figure S5. Surface tension experimental data (σ) of EAN at 25 °C of the present work (black solid triangles) and a compilation of that published near water content studied (w), with accurate measure of water content (solid) or not (open): [S10] (green dots), [S15] (yellow dots), [S17] (orange dots), [S26] (cyan dots), [S27] (red dots), [S48] (pink dots), [S50] (grey dots) and [S66] (dark green dots).

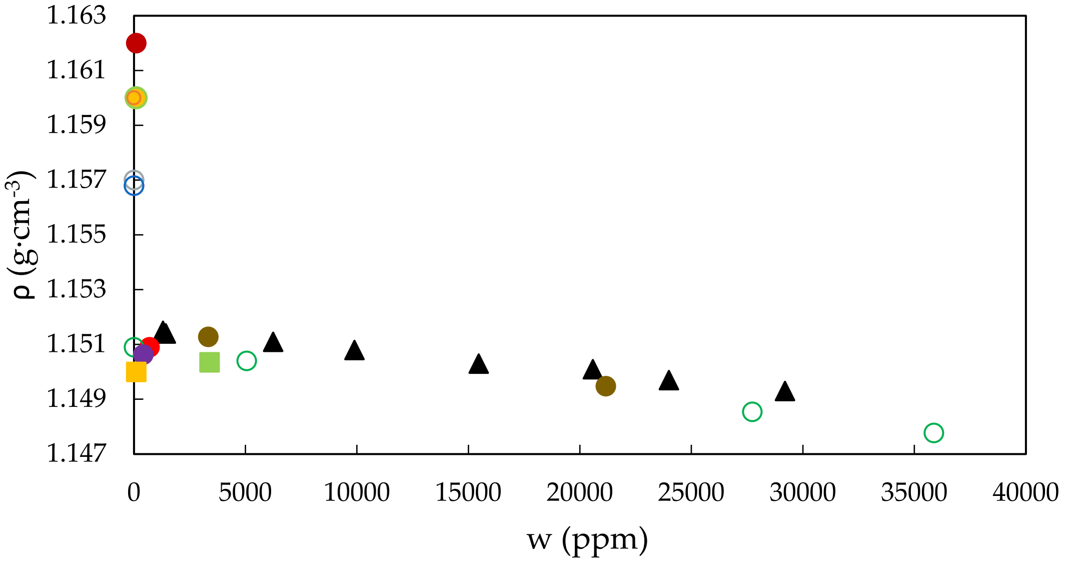

Figure S6. Density experimental data (ρ) of PAN at 25 °C of the present work (black solid triangles) and a compilation of that published near water content studied (w), with accurate measure of water content (solid) or not (open): [S5] (green dots), [S6] (yellow dots), [S7] (orange dots), [S11] (cyan dots), [S14] (red dots), [S44] (pink dots), [S45] (grey dots), [S46] (dark green dots), [S50] (brown dots), [S51] (blue dots), [S55] (dark red dots), [S57] (purple dots), [S61] (green squares) and [S62] (yellow squares).

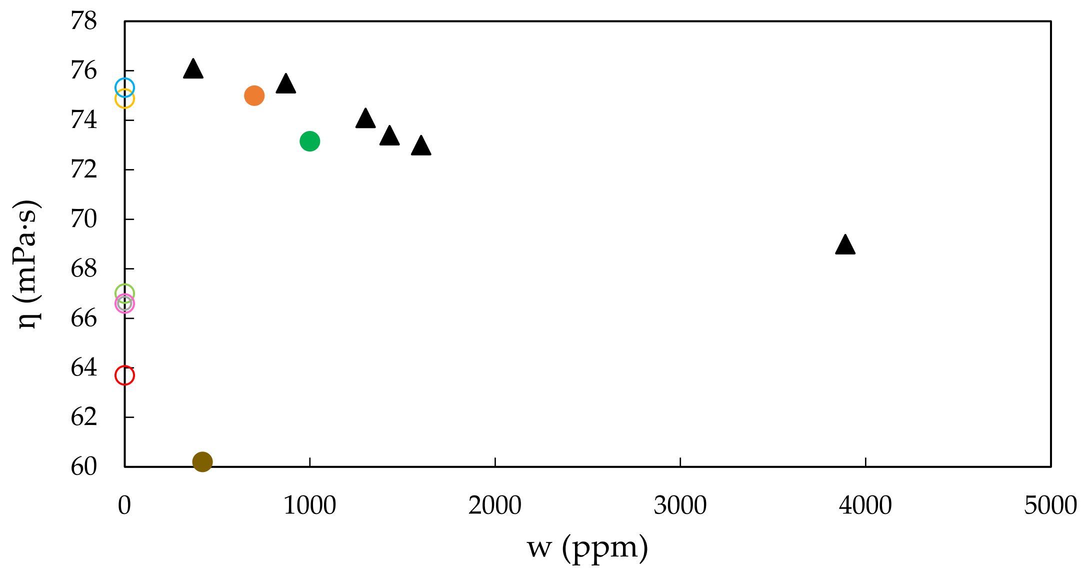

Figure S7. Viscosity experimental data (η) of PAN at 25 °C of the present work (black solid triangles) and a compilation of that published near water content studied (w), with accurate measure of water content (solid) or not (open): [S11] (green dots), [S13] (yellow dots), [S14] (orange dots), [S15] (cyan dots), [S44] (red dots), [S45] (pink dots), [S51] (grey dots), [S53] (dark green dots) and [S57] (brown dots).

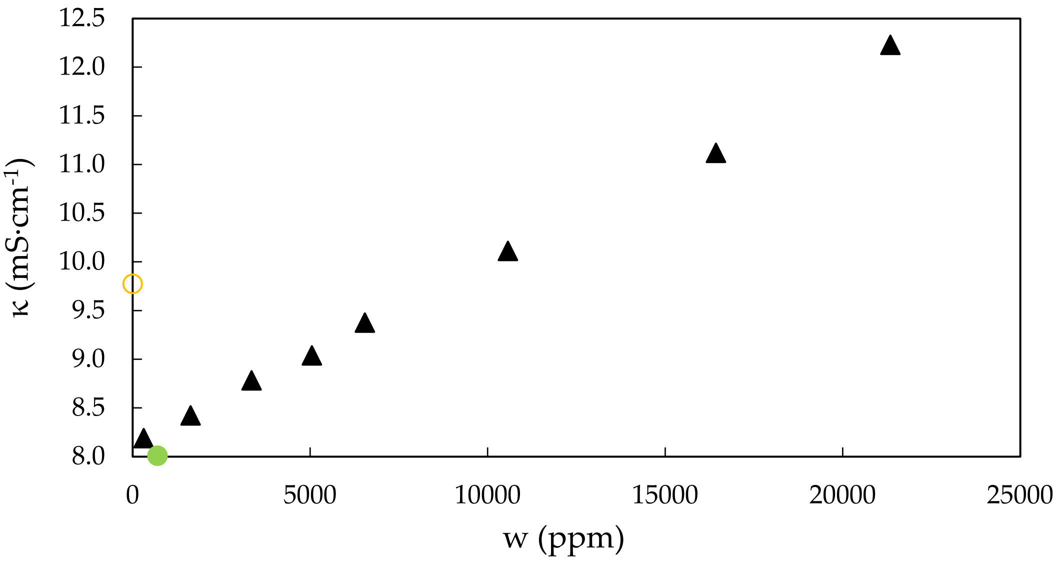

Figure S8. Electrical conductivity experimental data (κ) of PAN at 25 °C of the present work (black solid triangles) and a compilation of that published near water content studied (w), with accurate measure of water content (solid) or not (open): [S14] (green dots) and [S15] (yellow dots).

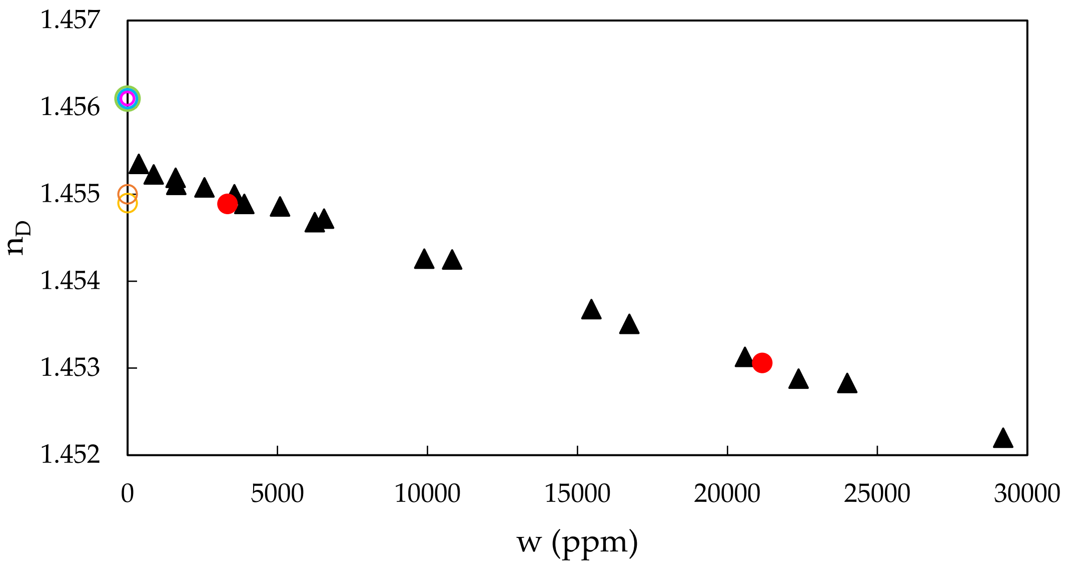

Figure S9. Refractive index experimental data (n_D) of PAN at 25 °C of the present work (black solid triangles) and a compilation of that published near water content studied (w), with accurate measure of water content (solid) or not (open): [S11] (green dots), [S15] (yellow dots), [S44] (orange dots), [S45] (cyan dots), [S50] (red dots) and [S51] (pink dots).

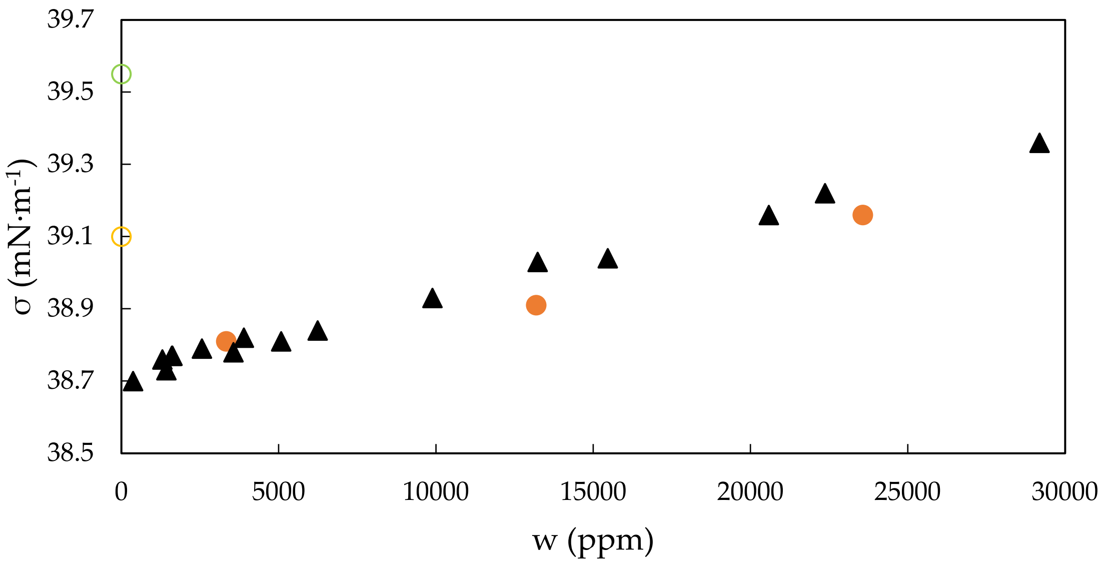

Figure S10. Surface tension experimental data (σ) of PAN at 25 °C of the present work (black solid triangles) and a compilation of that published near water content studied (w), with accurate measure of water content (solid) or not (open): [S15] (green dots), [S48] (yellow dots) and [S50] (orange dots).
